# Biases in *Drosophila melanogaster *protein trap screens

**DOI:** 10.1186/1471-2164-10-249

**Published:** 2009-05-28

**Authors:** Jelena Aleksic, Ranko Lazic, Ilka Müller, Steven R Russell, Boris Adryan

**Affiliations:** 1 Cambridge Systems Biology Centre, University of Cambridge, Cambridge, CB2 1QR, UK; 2 Department of Genetics, University of Cambridge, Cambridge, CB2 3EH, UK; 3 Aker Subsea Ltd, Controls R&D Department, Aberdeen, AB21 0NA, UK; 4 Current address: BioFocus DPI, Chesterford Research Park, Saffron Walden, CB10 1XL, UK

## Abstract

**Background:**

The ability to localise or follow endogenous proteins in real time *in vivo *is of tremendous utility for cell biology or systems biology studies. Protein trap screens utilise the random genomic insertion of a transposon-borne artificial reporter exon (*e.g*. encoding the green fluorescent protein, GFP) into an intron of an endogenous gene to generate a fluorescent fusion protein. Despite recent efforts aimed at achieving comprehensive coverage of the genes encoded in the *Drosophila *genome, the repertoire of genes that yield protein traps is still small.

**Results:**

We analysed the collection of available protein trap lines in *Drosophila melanogaster *and identified potential biases that are likely to restrict genome coverage in protein trap screens. The protein trap screens investigated here primarily used *P*-element vectors and thus exhibit some of the same positional biases associated with this transposon that are evident from the comprehensive *Drosophila *Gene Disruption Project. We further found that protein trap target genes usually exhibit broad and persistent expression during embryonic development, which is likely to facilitate better detection. In addition, we investigated the likely influence of the GFP exon on host protein structure and found that protein trap insertions have a significant bias for exon-exon boundaries that encode disordered protein regions. 38.8% of GFP insertions land in disordered protein regions compared with only 23.4% in the case of non-trapping *P*-element insertions landing in coding sequence introns (p < 10^-4^). Interestingly, even in cases where protein domains are predicted, protein trap insertions frequently occur in regions encoding surface exposed areas that are likely to be functionally neutral. Considering the various biases observed, we predict that less than one third of intron-containing genes are likely to be amenable to trapping by the existing methods.

**Conclusion:**

Our analyses suggest that the utility of *P*-element vectors for protein trap screens has largely been exhausted, and that approximately 2,800 genes may still be amenable using *piggyBac *vectors. Thus protein trap strategies based on current approaches are unlikely to offer true genome-wide coverage. We suggest that either transposons with reduced insertion bias or recombineering-based targeting techniques will be required for comprehensive genome coverage in *Drosophila*.

## Background

Genetic trapping experiments have a long-standing history in *Drosophila *functional genomics. The classic "enhancer trap" screens utilised *P*-element-mediated insertion of the *E. coli lacZ *gene to facilitate relatively unbiased discovery of tissue-specifically expressed genes and enhancers at a genome-wide level [1-3]. In recent years, additional transposable elements such as *piggyBac *[4] or *Minos *[5] have found their way into research applications. Together, this set of transposons provides excellent tools for genome-wide genetic screens. While *P*-elements and *piggyBac *show some positional preferences towards insertion at the 5' regions of target genes, and thus have biased coverage with respect to the genome [4,6], *Minos *is reported to show a much more random genomic distribution of insertions [5] and may be more suited to genome-wide screens.

The introduction of the green fluorescent protein (GFP) in research applications opened new avenues for enhancer trapping, since the expression of a fluorescent reporter can be directly observed and detection does not require an enzymatic reaction [7]. A more recent advance in the gene trapping approach is the "protein trap" screen, which aims to create GFP-tagged versions of endogenous proteins under the control of the genes' native regulatory sequences [8-11]. As is evident from the comprehensive recombination-based efforts in yeast, tagging endogenous proteins in vivo can have tremendous utility for genomics, cell biology and systems biology studies [12]. Protein tagging is likely to be of even greater untility in metazoans where many different cell types are present.

Protein trap screens utilise artificial reporter-encoding exons to generate fluorescent fusion proteins by random integration into the genome. The reporter is usually a GFP variant, flanked by splice acceptor/donor sites, and carried within a transposable element vector. Integration of the transposon within an intron in the correct orientation results in the transcription and subsequent splicing of the trapping exon into the mature mRNA of the targeted gene. If the trapping exon is in-frame with coding sequence of the host protein, a functional GFP-tagged version of the protein may be produced. A comprehensive screen obviously requires vectors carrying targeting exons in all three reading frames, but even when multiple vectors are employed the isolation of bona fide protein traps is a relatively rare event.

Following the pioneering work of Morin et. al. [8], the results from a variety of *Drosophila *protein trap screens have been published [8,10,11,13]. In total, these studies have screened close to 80 million individual embryos or larvae, with only a small fraction of these generating lines that express the GFP tag. The FlyTrap database [13] reports 1,522 fluorescent lines generated from the three largest protein trap screens performed to date [8,10,11]. Mapping of their insertion coordinates to Release 5.3 gene annotation shows that these represent 271 unique genes tagged with protein traps located within introns separating coding exons. With the fly genome containing approximately 14,000 protein-coding genes, the screens have hit less than 2% of known fly genes. This is far from genome-wide coverage, clearly a desirable goal for comprehensive functional genomics studies. The restricted success of protein trap screens is especially surprising given that approximately 11,600 *Drosophila *genes contain introns. Thus, in principle, approximately 80% of fly genes are accessible to protein trapping. Interestingly, although the overall number of unique lines generated in the different screens is relatively small, there is considerable overlap in the tagged genes recovered by the individual screens.

The low efficiency and high degree of overlap between the published screens suggests that there are limitations to the protein trap strategies currently in use. Here we attempt to identify and quantify these limitations, and suggest future strategies that may increase the repertoire of trapped genes. We considered a number of potential factors that could bias protein trap screens, including transposable element integration hotspots, gene architecture, gene expression and protein structure. We constructed a probability model that we used to predict a set of target genes with a high likelihood of successfully receiving a protein trap insertion. Our model predicts that approximately 800 of the genes encoded in the fly genome are permissive for *P*-element based protein trapping and of these, 264 genes have already been tagged in previous studies (with *P*-element or *piggyBac*, in previously published and novel screens). A similar analysis based on data from a more limited set of protein traps generated with *piggyBac *vectors estimates approximately 3,100 genes are permissive targets with this transposon, and about 2,800 of these have not yet been tagged in previous studies. Comparing the predictions for both transposons we find that most potential as yet untagged *P*-element targets are also good *piggyBac *targets (448 out of 536 potential *P*-element targets). Due to the apparent importance of transposon insertion bias, it is likely that a transposable element such as *Minos*, which exhibits a more random insertion preference [5], may be a better vector for future random protein trap screens. Ultimately, it is likely that recombination or recombineering -based gene targeting techniques will need to be employed to achieve comprehensive coverage of the fly genome.

## Results and Discussion

### The minority of GFP trap lines are real protein traps

We retrieved insertion data for 1,522 previously described protein trap lines from the FlyTrap database [13]. The genomic insertion coordinates were available for 1,471 of the lines and these were taken forward for further analysis. The insertion sites from FlyTrap were translated to *Drosophila *genome Release 5 coordinates and mapped with respect to the Release 5.3 gene annotations taken from FlyBase. The exon boundary and coding sequence information was obtained for each transcript of the relevant genes and, where available, the relevant frame information from the original protein trap screen was utilised. Together, these data established a catalogue of protein trap target genes based on the most recent genome sequence and annotation releases. The compiled data are available [see Additional file 1] with a summary provided in Table 1.

**Table 1:** Annotation changes after mapping to Release 5.3 for the 1,471 FlyTrap lines with available coordinates.

		**original FlyTrap annotation** (see *flytrap.med.yale.edu *for definitions)					
		
		Protein (498)	Protein? (117)	Enhancer (389)	Enhancer? (334)	Novel (133)	**total**
**Release 5.3 annotation**	CDS intron	373	4	35	28	9	449
	
	other genic region	34	60	190	144	48	476
	
	intergenic region	91	53	164	162	76	546

The updated insertion coordinates were mapped to genes from FlyBase Release 5.3, and the insertion annotations were updated accordingly. The mapping of a small number of lines was ambiguous due to the insertion being in various features of different transcripts and/or different nested genes – in the cases where an intron between coding sequence was hit, the line was classified as 'CDS intron', regardless of the other features hit concurrently. Some genes are represented by multiple lines, so the 449 fly lines hitting CDS introns in fact amount to 271 unique genes hit.

**Figure 1:**
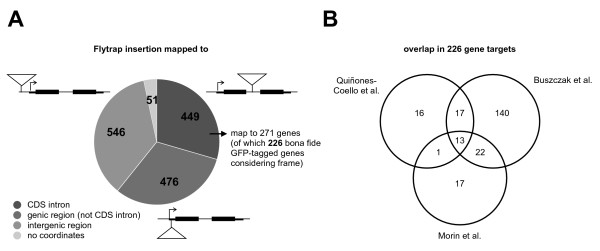
**GFP protein-trap insertions**. **A) **Mapping insertions to gene features: the 1,522 insertions reported in the FlyTrap database were mapped to Release 5.3 of the *D. melanogaster *gene annotation, with the results relative to gene features shown. **B) **Overlap in protein trap targets: the 226 protein trap targets with matching frame information reported in FlyTrap originate from three large-scale screens and there is considerable overlap between the gene sets obtained.

A canonical protein trap is defined as an insertion residing in an intron within the coding sequence of a gene that, after splicing, allows the translation of the GFP reporter in frame with the endogenous polypeptide. The recovery of enhancer traps in protein trap screens has been previously observed and determined to be due to the presence of an upstream start codon. The start codon is within the transposase gene of the protein trap vector itself [10], but has also been reported to result from a splicing event from the non-coding strand of the mini-*white *marker present in the vector [11]. The updated Release 5 coordinates and gene annotations confirm that bona fide protein traps constitute the minority of the 1,522 reported lines (Figure 1A). Of the 1,471 fly lines for which coordinates were available, 546 are insertions entirely outside gene regions and of the 925 remaining lines that do insert within a gene model, only 449 are found in introns between coding exons. These 449 insertions tag a total of 271 unique genes, reducing to 226 genes if frame information is taken into account. The 1,022 insertions in places other than the introns within coding sequence are considered to be enhancer trap type insertions rather than protein traps. 429 of the FlyTrap lines have insertions targeting gene regions outside their coding sequence. Interestingly, only 6 of such insertions are located in the 3' UTR of genes with the remainder 5' to the gene start codon. While this suggests that it is indeed likely that the *P*-element promoter and translational start sites facilitate enhancer trapping, some of the traps may be located in introns associated with unannotated 5' exons and thus may represent bona fide protein traps.

### Insertion biases of protein trap insertions

Approximately 11,600 protein-coding genes in the fly genome have at least one intron and therefore 80% of the *Drosophila *gene repertoire is in principle amenable to GFP-tagging. In practise, the fraction of recovered protein traps is clearly much lower, indicating that there are preferred target genes. This becomes evident when the total of 226 GFP-tagged proteins found in the three published large-scale screens are compared (Figure 1B). While the largest screen [11] successfully tagged a number of new proteins (140, i.e. 72.9% of the genes found do not feature in the other screens), most of the proteins tagged in the smaller screens are recovered in multiple studies. 68% (36 genes) of the protein traps reported by Morin et al. [8] and 66% (31 genes) of those reported by Quiñones-Coello et al. [10] were also recovered in one or both of the other screens.

### Transposable element hotspots introduce the greatest bias in protein trap screens

The *P*-element constructs of Morin et al. [8] were used in the two other screens reported in FlyTrap and lines derived with these vectors represent the majority of the insertions reported in the database (81% of the lines in FlyTrap). The remaining insertions were generated with a *piggyBac *vector (see below). To explore the possibility that *P*-element insertion preference biases the recovery of protein traps, we compared the repertoire of genes recovered in the protein trap screens with all of the *P*-element insertions reported in FlyBase. Twelve genes were independently recovered using *P*-elements in all three protein trap screens and we found an average of 20 other *P*-element insertions in each of these genes (min 3, max 61). These 12 genes alone are covered by 64 independent lines reported in FlyTrap, accounting for 4.2% of all the lines in the database and over 13% of coding sequence insertions. This observation suggests that there may be significant bias due to *P*-element insertion hotspots. Noteworthy, amongst the 12 targets shared between all protein trap studies is CG9894, which accounts for 10% of all "KG" (Karpen Genome) lines recovered in the Gene Disruption Project [14].

**Figure 2:**
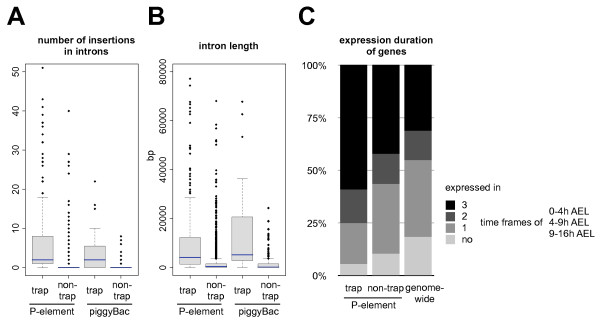
**Insertional biases**. **A) **Number of transposable element insertions in introns: introns of canonical GFP-tagged genes were divided into 'trap' if they carried the GFP-trap insertion and 'non-trap' if not. Targets were independently analysed for *P*-element and *piggyBac *insertions. While the 'trapped' introns generally show large numbers of previously reported *P*-element or *piggyBac *insertions, the 'non-trapped' introns show significantly lower values. **B) **Intron length: genes with previous *P*-element or *piggyBac *insertions were divided into 'trapped' or 'non-trapped' depending on the existence of a GFP-trap insertion. The average length of the introns hit by *P*-element traps is 9.2 kb and is 18.2 kb for *piggyBac *targets, which is significantly higher than the 1.9 kb and 1.8 kb average for other non-trapped introns in the same genes. **C) **Expression duration of genes: embryonic gene expression was divided into three roughly equivalent time frames. GFP-trap targets show prolonged gene expression compared to other genes susceptible to *P*-element-insertion (data for *piggyBac *not shown). The small number of genes with no embryonic expression originated from the pilot screen performed on L1 larvae.

For all remaining protein trap targets generated using *P*-element vectors, the insertion location is strongly biased. Of the 248 genes containing *P*-element GFP trap insertions into coding sequence introns (irrespective of frame), 237 (95.6%) have other *P*-element insertions and only 4 genes have no previously reported transposon insertions. The frequency of *P*-element insertions in genes isolated in the protein trap screens is significantly higher than observed within the approximately 7,000 protein-coding genes that have reported *P*-element integrations (χ^2 ^test, p < 10^-4^). In addition, within the protein trap tagged genes, the integration hotspots are localised within the tagged intron since these have, on average, 6.29 other *P*-element insertions. Other introns within tagged genes have, on average, only 0.67 *P*-element insertions (Figure 2A; p-value < 2.2 × 10^-16^, Wilcoxon test).

Two of the published protein trap screens [10,11] also used *piggyBac*-based vectors, because of their reported preference for AT-rich integration sites such as introns [4]. These pilot screens generated 234 lines of which 45 insertions result in protein traps in 33 unique genes. Interestingly, the same bias towards previously isolated transposon targets is also found in the *piggyBac *set (Figure 2A) with 31 out of 33 genes containing previously reported transposon insertions: 24 of the 33 have other *piggyBac *insertions. A total of 144 previously reported *piggyBac *insertions are associated with the protein trapped genes recovered in the *piggyBac *screens (average of 4.36 insertions per gene): a comparable level to that observed in *P*-element screens. The high frequency of other transposable element insertions associated with genes recovered in the protein trap screens indicates that these genes, and more specifically their tagged introns, are genomic hotspots for transposon insertion.

### GFP traps selectively target longer introns

Due to the much larger number of *P*-element protein trap lines reported in FlyTrap, we concentrate the majority of our further analysis on the differences between genes generally susceptible to *P*-element integration and genes recovered in the protein trap screens, to identify factors that may influence recovery of protein traps. The average length of the 248 genes successfully tagged by *P*-element protein traps is 19.5 kb, significantly higher than the 9.7 kb average size of genes with other types of *P*-element insertion and the 3.3 kb average size of the genes in the genome without *P*-element insertions (p < 2.2 × 10^-16^, Kruskal-Wallis test). The dramatic difference in gene length is mainly due to differences in cumulative intron length. The mean cumulative intron length for protein trap genes is 17.9 kb, compared to 8.8 kb for other genes with other *P*-element insertions (p < 2.2 × 10^-16^, Wilcoxon test). Two factors contribute to the higher cumulative intron length (Figure 2B): protein trap targets have a larger number of introns (7.08 versus 5.56, p < 2.2 × 10^-16^, Wilcoxon test) and the individual introns are longer (3.1 kb vs 1.6 kb, p < 2.2 × 10^-16^, Wilcoxon test) compared to other genes with non-protein trap *P*-element insertions. The protein trap insertions preferentially land in longer introns since trapped introns are, on average, 9.2 kb long, significantly longer than the 1.9 kb average for other introns within the same genes (p < 2.2 × 10^-16^, Wilcoxon test). These numbers confirm trends already recognised in the pilot studies by Morin et al. [8], where for the candidate gene set the average length of targeted introns was reported larger than 2.5 kb and thus significantly larger than the genomic average. This observation provides one explanation for how protein trap targets differ from other genes susceptible to *P*-element integration since they contain larger landing spaces for the GFP-encoding exon. It is noteworthy that a similar trend is also observed for introns trapped with *piggyBac *vectors (Figure 2B) since the introns hit by *piggyBac *are on average 18.2 kb long, more than 13 times larger than the genome-wide intron length average of 1.33 kb (p < 2.2 × 10^-16^, Wilcoxon test).

### Protein trap targets show broad and persistent gene expression

Host gene expression has a direct influence on GFP detection in protein trap screens. Detection success largely depends on two parameters: signal strength (the fraction of GFP-positive cells in the animal and how brightly each cell fluoresces) and expression duration (a transiently expressed gene is less likely to be detected). In general, protein trap screens select individual GFP-positive progeny generated in a particular cross from a much larger background of GFP-negative individuals. The screens generally take a momentary snapshot of the population and thus, in order to be recovered, a tagged protein must be expressed at detectable levels when the selection is made. Morin et al. screened first instar larvae manually with a dissecting microscope, while both of the large-scale screens utilised an automated embryo sorter. Good examples of the importance of expression timing were observed by Quiñones-Coello et al. [10]. They found that the *Dek *gene (highly expressed in first instar larvae) was independently hit 25 times by Morin et al. [8] but never in the embryo based screen, and vice versa with *extra macrochaetae *(*emc*), which was detected 11 times by Quiñones-Coello et al. but not by Morin et al. However, even within the period of expression in the embryo, our analysis quantified biases linked to expression.

To assess the contribution of host gene expression to protein trap recovery, spatio-temporal gene expression was analysed, where available, for genes containing *P*-element protein trap insertions. For this analysis, *Drosophila *embryonic development was broadly divided into three roughly equivalent time frames (I = stage 1–8, 0–4 h After Egg Laying (AEL); II = stage 9–12, 4–9 h AEL; III = stage 13–16, 9–16 h AEL). Using data from the BDGP in situ database [15], we determined in how many of these three time frames each target gene is expressed. Expression information was available for 160 out of the 248 protein-trapped genes. The set of GFP-tagged genes is significantly biased towards expression in two or three time frames (75%), whereas only slightly more than 50% of other genes with non-trap *P*-element insertions show prolonged expression (Figure 2B; χ^2 ^test, p < 10^-3^). This is expected, since the longer a gene is expressed the greater the likelihood that it will be detected in a screen. The expression of successfully tagged genes is not only persistent, but also tends to occur in broad domains and comparatively large tissues. A striking 121 of the 160 genes (75.6%) with expression information are annotated as being maternally contributed where, in most cases, the tagged protein is expected to be distributed throughout the embryo. This is an interesting observation because in both large-scale screens, *P*-elements were mobilised in males and thus the initial selection could not be based on maternal contributions. Many tagged genes also exhibit ubiquitous expression in later embryonic stages (68; 42.5%). Other relatively large tissues that are enriched for protein-trapped genes include the embryonic brain (57; 35.6%), the ventral nerve cord (55; 34.4%) and various tissues of mesodermal origin. This suggests that, if the GFP signal follows mRNA expression, most GFP-tagged genes are expressed in at least 20% of the total embryonic cell population. Genes tagged using a *piggyBac *vector display the same trends. Although expression data is available for only 21 of the 33 tagged genes, these tend to be broadly expressed, on average in 2.57 of the 3 stages. The spatial and temporal data taken together confirm the assumption that there is a detection bias for genes with persistent and broad embryonic expression.

### Introns at exon-exon boundaries that map to unstructured protein regions are frequently hit

A functional target protein can only be obtained if integration of the 28 kDa GFP polypeptide occurs in places where it is not structurally constrained. In cases where GFP insertion into a protein disrupts the folding or is otherwise detrimental, we expect that the tagged protein will be degraded and thus such lines are unlikely to be recovered in screens. In fact, 36% of the canonical protein trap lines studied by Quiñones-Coello et al. [10] were homozygous lethal, of which two thirds were associated with the GFP-exon by genetic complementation experiments. In general, therefore, the recovered protein trap lines provide a snapshot of insertions that are tolerated with respect to protein structure and folding. The crystal structure of GFP indicates that its N- and C-termini are in relatively close proximity [16,17], suggesting that GFP may be able to act as a linker between individual protein domains or can be inserted at surface exposed areas within domains without altering the overall fold of the targeted protein.

**Figure 3:**
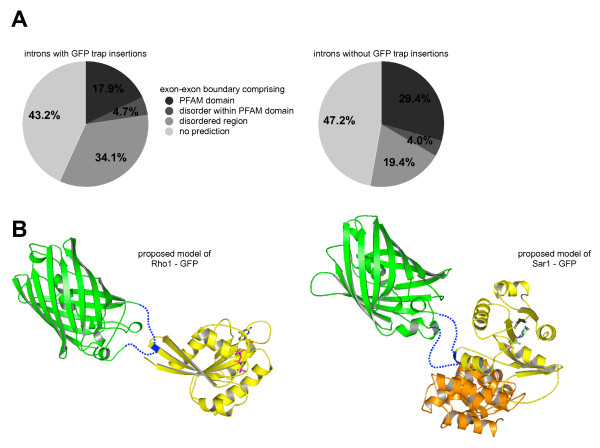
**Structural constraints on GFP polypeptide insertion**. **A) **GFP trap hotspots: the left chart plots the relative frequency of GFP insertions in introns between exon-exon boundaries comprising predicted structural domains, regions of intrinsic disorder or unclassified regions. The right chart plots un-trapped introns from the same genes and shows a reduction in the intrinsically disordered category. **B) **Consequences of GFP insertions: in cases where GFP insertions fall into predicted structural domains, mapping of domain sequence to known structures of proteins of the same fold shows that it is mostly surface exposed areas that are affected. In both examples, the overall fold of the GFP target domain is unlikely to be affected by the insertion. Note that the linker residues and the C-terminus of GFP (blue dotted lines) are predicted to be highly flexible. The displayed examples show only one possibility for how the GFP domain is structured relative to the host protein.

We classified the 1,906 protein regions encoded around exon-exon boundaries in the GFP-tagged transcripts, of which 296 are interrupted by the GFP insertion. We found a statistically significant preference (χ^2 ^test, p < 10^-3^) for structurally disordered/unfolded regions as predicted by DISOPRED2 [18] (38.9% for tagged introns vs 23.4% for non-tagged introns), whereas unclassifiable regions are hit at similar rate (43.2% vs 47.2%). Domains predicted in the PFAM protein families database [19], excluding those overlapping with disordered regions, appear to be avoided (17.9% vs 29.4%; Figure 3A). Interestingly, manual inspection of three-dimensional structures of directly hit PFAM domains showed that the GFP insertion sites are located mostly at the surface, leaving the overall fold of the targeted domain unchanged (Figure 3B). In the example of Rho1-GFP, the GFP insertion (shown in green, PDB ID 2hfc) into the Ras domain (yellow, PDB ID 3rab, 29.2% sequence identity) takes place at a solvent exposed β-strand (blue) distant from the GTP binding site. Sar1 is a protein of the Arf family and the example structure shows the small GTP binding protein Arf1 (yellow) in complex with its activator, GEF-ARNO (orange, PDB ID 1r8s, 34.6% sequence identity). Sequence mapping places the GFP insertion in a solvent exposed loop remote from both nucleotide and activator binding sites. In both examples, the active domains of the targets in question remain unaffected by the GFP insertion.

In the course of this analysis we also identified a variety of correlations between intron features, as well as between intron features and structural features encoded at the respective exon-exon boundaries (data not shown). For example, the mean length of introns at unstructured boundaries is approximately 1.5 kb, more than twice as long as the mean of those interrupting PFAM domains (p < 2.2 × 10^-16^, Wilcoxon test). The preference of *P*-element insertions for longer introns suggests that the identified bias for disordered/unfolded regions may be a result of the general insertion bias, and not of detrimental effects to protein structure. While neither of these two possibilities can be ruled out, we observed that the 316 introns at exon-exon boundaries in GFP-tagged genes that do not have a protein trap insertion but do have a non-trap insertion show essentially the same characteristics as those without any transposon insertion (comprising PFAM domain: 27.5%, disorder: 21.8%, PFAM domain with disorder: 5.1%). This observation suggests that insertion bias alone is not sufficient to explain the GFP trap preference for unstructured regions.

It is noteworthy that protein-trapped genes often exhibit a number of splice variants, some of which are not affected by the trap insertion: of 248 GFP-tagged genes, 102 (41.1%) are predicted to have additional unaffected transcripts. It should be noted that the impact of GFP insertion on protein structure cannot fully be assessed due to the lack of experimental data and because only 20% of the primary protein sequence in the fly possesses reliable structural predictions (BA, unpublished observations).

### The majority of likely targets have already been hit

Morin [20] estimated that only a couple of hundred loci are likely to be successfully targeted by *P*-elements, and that different vectors will soon be required. Based on our in-depth analysis from the data that have since become available, we set out to more precisely quantify this prediction. Taking the above observations together we identified insertion biases, gene expression and protein structural constraints as likely limiting factors for the success of protein trap screens. A cumulative score representing the likelihood of a successful insertion was calculated independently for each intron. The parameters used for the prediction included the number of previous *P*-element hits, the length of the intron, the presence of intrinsic disorder in the protein region encoded around the exon-exon boundary, and, where available, the expression data for the gene in question (see Materials and Methods for model building and benchmarking).

**Figure 4:**
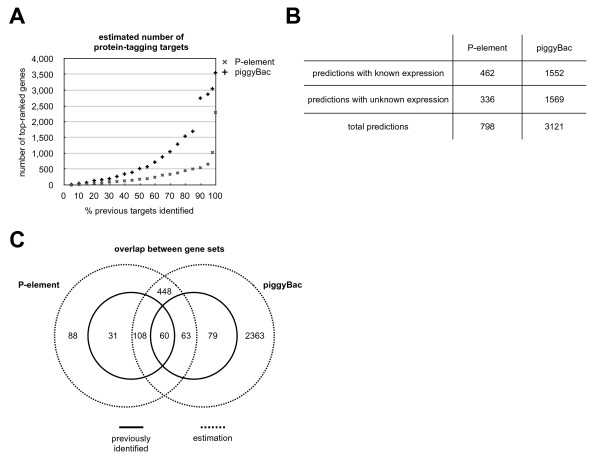
**Prediction of protein-trap targets**. **A) **Estimated number of protein-tagging targets for genes with known expression: genes were ranked according to their highest scoring introns. The sets of predicted genes were then compared to the reported GFP-trap target genes to determine an overlap. The graph shows the number of top-ranked genes considered in order to recover 10%, 20% etc of previously known GFP-trap target genes. **B) **The predicted numbers of possible targets for *P*-element- and *piggyBac*-based GFP trap screens. The numbers were first derived separately for genes with known and genes with unknown expression, and the total prediction is the sum of the two. The predictions show the number of top genes predicted by the model required for an 80% coverage of previously known protein trap insertion targets. **C) **Overlap between P-element and *piggyBac *targets: the inner circles of the Venn diagrams (solid lines) represent the numbers of reported *P*-element and *piggyBac *gene hits, the outer circles (dotted lines) represent the numbers of estimated gene targets derived from our model. There is an overlap between both the reported and the predicted targets. Only reported genes that have been successfully predicted by the theoretical model are shown in this diagram.

The model was used to generate a list of high probability candidate genes from the set of all genes with expression information. We then asked, how many of the top-ranked genes from this list need to be accepted to recover the *P*-element-based protein trap insertions listed in FlyTrap for which gene expression data is available (Figure 4A). For example, to identify 80% of the genes in FlyTrap we need to accept 462 predicted targets. Of these 462 genes, only 34% have been identified in protein trap screens. Analogously, the analysis was repeated for genes with no expression information considering only the other three model parameters. In order to identify 80% of the FlyTrap genes with unknown expression, 336 genes of unknown expression need to be accepted (data not shown). Taken together, 798 high-ranking genes recover 80% of the *P*-element-based GFP-tagged genes (Figure 4B). We also applied our estimation to data from a set of *piggyBac*-based protein traps, combining the 234 lines from FlyTrap with 504 lines from an ongoing screen at the University of Cambridge (Daniel St Johnston, Kathryn Lilley and Steven Russell, unpublished results). Under the assumption that similar biases apply to *piggyBac*-based protein trap screens, we predict more than 3,100 potential target genes to recover 80% of lines already identified (Figure 4B). This suggests that there is a larger set of genes still amenable to protein-tagging using *piggyBac *compared with *P*-element vectors. The respective overlap between previously identified GFP-tagged genes with estimations based on 80% recovery for both transposable elements (for genes where expression data is contained in the BDGP in situ database) is shown in Figure 4C. This indicates that *P*-elements are reaching the end of their useful life as protein trap vectors, whereas screens utilising *piggyBac *are likely to still have potential for yielding more trapped genes. However, the analysis also suggests that the utility of *piggyBac *vectors is also finite and other methods will have to be developed to achieve genome-wide coverage.

## Conclusion

Transposable element-based protein trap screens have strong underlying biases, largely dependent on which transposon vector is used. While *P*-element based screens are predicted to yield additional new inserts, this is likely to be at very low efficiency, since most high-probability targets have already been hit. Studies employing *piggyBac*-based vectors appear to be more promising, with potentially 5 times as many targets expected compared with the *P*-element-based approach. However, given the significant overlap between the predicted target genes obtainable with both vectors and the existence of restraints on their insertion location, transposable element-based approaches are likely to reach the end of their utility in the near future. An assessment of the possibility of using a different transposon vector such as *Minos*, which exhibits a more random insertion pattern [5], is therefore recommended. At present there are insufficient data for us to predict the number of *Minos *targets in the *Drosophila *genome. In the long run, to achieve true genome-wide coverage or to target functionally important gene classes such as transcription factors, targeting vectors utilising homologous recombination will have to be designed for protein trapping. Structural restraints will still apply in such cases and we note that optimal exon-exon boundaries encoding regions with local disorder are only predicted for approximately half of the intron containing genes in *Drosophila*. The use of homologous recombination methods combined with careful construct design, taking into account the underlying limitations of protein trap screens, could be a potential route for attaining better genome coverage. Alternatively, a recombineering-based approach in *Drosophila *has recently been developed [21], which allows tagging of specific genes with a variety of functional moieties, and is amenable to high-throughput applications. While protein tagging recombineering studies have not yet been reported, it is likely that this method may well lead to more complete genome coverage.

## Methods

### Data compilation

GFP-trap insertion coordinates were obtained from the FlyTrap database [13], with the exception of the Morin et al. (2001) frame information, which was provided by Xavier Morin (personal communication). The original genomic coordinates from FlyTrap were converted to *Drosophila *genome Release 5 using the UCSC LiftOver tool . Coordinates of genetic features and sequence data were retrieved from the UCSC Genome Browser database [22]. Additional file 1 contains a compilation of all relevant data, along with a brief analysis of target gene overlap between the different genome releases.

### Data analysis

Transposable element integration coordinates and spatio-temporal gene expression information were retrieved from FlyMine [23]. Disordered protein regions were predicted at 5% FDR using DISOPRED2 [18] and PFAM domains were assigned using the pipeline described in [24].

All data analysis was performed using custom-written Perl scripts and statistical tests were performed with R.

### Prediction of genes susceptible to protein tagging

We developed a measure for the likelihood that an intron will be a successful protein trap target, based on:

1) Previously identified *P*-element integrations.

2) Intron length.

3) Target gene expression

4) Protein structural constrains of the surrounding exon-exon boundary.

Using these criteria, a score was derived for each intron within a coding sequence encoded in the *Drosophila *genome, leading to a list of potential candidate genes. The strategy was developed using the *P*-element-based GFP trap insertions for modelling and benchmarking, but was then also applied to the genes recovered in *piggyBac*-based screens.

Individual scores were derived as follows:

1) TE-score: the probability that an intron will be susceptible to insertion of the respective transposable element (TE) was calculated as the ratio of GFP-tagged introns with exactly *n *previous TE insertions divided by the total number of introns with exactly *n *previous TE insertions. The minimum number of data points used per interval was 20. In general, the interval used was 1. However, because of the scarcity of the data for high values of *n*, data points for increasing ranges of *n *were binned together by extending the interval symmetrically (to *n+1 *and *n-1*) until the minimum of 20 data points was reached. The resulting ratios were fitted using non-linear least squares regression in Matlab v7.7, yielding the function *y *= *a *+ (1-*a*)·*tanh(b·x) *to describe the observed trend (with *a *= 0.18 and *b *= 0.44). The original data, the fitted line and the error graph are shown in Additional file 2, Figure A.

2) Length-score: a score based on intron length was devised by binning introns into 1 kb intervals, omitting the 20% shortest and longest introns to avoid extreme outlier values. The fitting function was determined as *y *= *9*·*10*^-6^·*x *+ *0.0677*. A plot of the ratios and fitted line is presented in Additional File 2, Figure B.

3) Expression-score: according to whether the gene is expressed in none, 1, 2, or all 3 time frames, it was assigned probabilities of 0, 1/3, 2/3 or 1, corresponding to the proportion of time the protein is likely to be present for detection.

4) Disorder-score: introns comprising exon-exon boundaries encoding disordered region receive a bonus. The probability of a GFP trap insertion was determined to be 0.305 for introns in disordered regions, and 0.147 for other introns.

To calculate the cumulative score for each intron, the four parameters were added together as *S *= *c*_1_·*TE-score + c*_2_·*Length-score + c*_3_·*Expression-score + c*_4_·*Disorder-score*, and the maximum score of all introns retained for each gene. The individual weights (c_1 _= 12; c_2 _= 1.5; c_3 _= 0.5; c_4 _= 0.5) were determined systematically by comparing the overlap of the top-scoring genes with the *P*-element-based GFP-tagged genes for all combinations of *c*_1–4 _from 0 to 12 in steps of 0.5. The resulting coefficients underline the strong influence that susceptibility to TE insertion has.

Training: intron scoring and benchmarking was only performed for genes with available BDGP expression data. The derived coefficients were then extrapolated to genes without expression information, under the assumption that these will behave the same with respect to the other parameters.

### Protein structures

The predicted structures of the GFP-host protein fusion domains were generated manually using the PFAM web site for sequence alignment, the RCSB Protein Data Bank [25] for structural information and Coot [26] to inspect the structures and construct the model structures. Two examples are presented in Figure 3B, with images generated by Pymol [27].

A representative structure for each target domain was chosen from the PDB, its sequence extracted and aligned against the hidden Markov model (HMM) of the domain family. Equally, the sequence of the GFP target domain was aligned against this HMM, which enabled us to assign the region of the GFP insertion with respect to the sequence of the reference structure. The 1.2 Å structure of GFP (PDB ID 2hfc, [28]) shows high flexibility in the C-terminal region with residues 231–235 not included in the model and the terminal residues 236–237 solely fixed in their position by crystallographic contacts. Consequently, these residues were not included in the GFP fusion models shown. The GFP structure reveals the relative proximity of its C- and N-terminus. Taking into account the intrinsic flexibility of the N- and C-terminus of GFP and the length of the linker residues introduced into the GFP-fusion gene products, the distance between the last structured residue in the GFP domain and the locus of the insertion in the target domain are likely to be in the range of 10–30 Å, allowing large flexibility for the relative orientation between these domains. The models displayed in Figure 3B only represent one possible arrangement.

## Authors' contributions

JA compiled the raw data, carried out most of the genome bioinformatics and modelling and wrote the paper. RL devised the modelling strategy. IM studied examples of GFP insertions on protein structure and carried out some of the structural bioinformatics. SR contributed the data from the *piggyBac*-based protein trap screen and wrote the paper. BA conceived the study, carried out most of the structural bioinformatics, and wrote the paper. All authors read and approved the final manuscript.

## Supplementary Material

Additional file 1 **Compiled data**. The table contains the original data (the FlyTrap line number, gene hit, coordinates, strand information, trap category and the original genome release that was used for mapping) followed by the updated information mapped to Genome Release 5 (the new coordinates, gene and transcript hit, feature hit, the original frame information and the frame the hit is supposed to be landing in according to the new mapping). To avoid ambiguities in mapping, separate entries were included for each transcript hit by the insertion. Click here for file

Additional file 2 **Determination of model parameters**. This figure shows the data used for obtaining the model parameters. **A) ***P*-element insertion bias: the graph shows the data for determining the likelihood of a GFP-trap hit based on the number of previous *P*-element insertions present within the intron (method described in detail in the Materials and Methods section). The line was fitted using a non-linear least squares regression. **B) **Intron length bias: the graph shows the hit probability calculated for each intron length (binned in 1 kb intervals). To reduce the impact of extreme outliers (mini-introns or very large introns), the top and bottom 20% of the data were removed. **C) **Disorder table: the chart shows the absolute number of GFP-trap hits documented in disordered and non-disordered protein regions along with the number of 'misses' for each (the introns in the same genes not hit by a protein trap). Click here for file
