# Supplementary figures and images for: Biases in Drosophila melanogaster protein trap screens
Source: BMC Genomics. 2009 May 28;10:249. doi: 10.1186/1471-2164-10-249 (PMC2695487; doi:10.1186/1471-2164-10-249)

**A**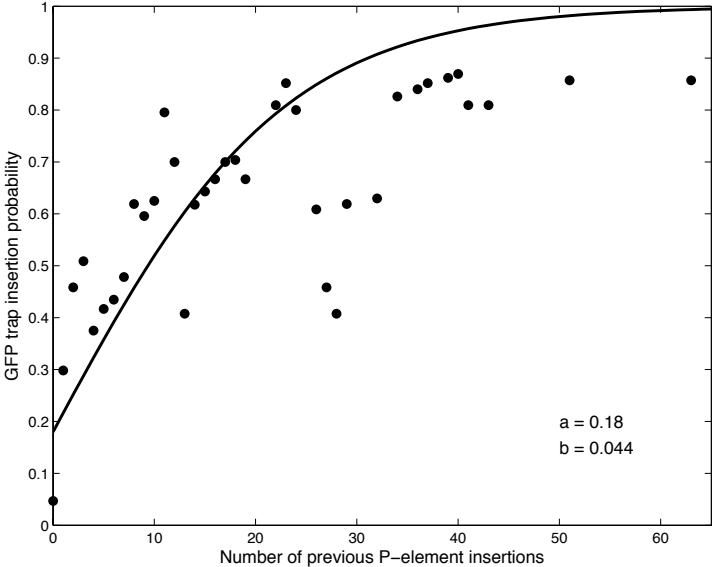**B**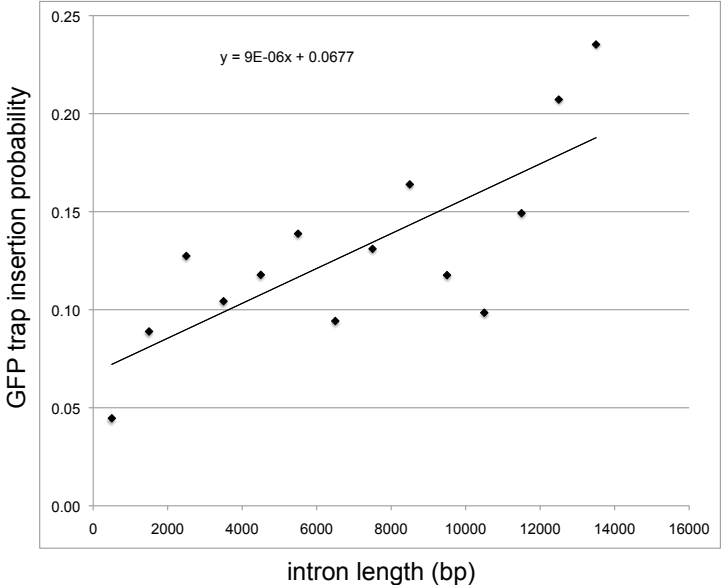**C**

|                           | disorder | no disorder |
|---------------------------|----------|-------------|
| intron with insertion     | 115      | 181         |
| introns without insertion | 377      | 1233        |
| probability               | 0.305    | 0.147       |

Supplement: Additional file 2 — Determination of model parameters. This figure shows the data used for obtaining the model parameters. A) P-element insertion bias: the graph shows the data for determining the likelihood of a GFP-trap hit based on the number of previous P-element insertions present within the intron (method described in detail in the Materials and Methods section). The line was fitted using a non-linear least squares regression. B) Intron length bias: the graph shows the hit probability calculated for each intron length (binned in 1 kb intervals). To reduce the impact of extreme outliers (mini-introns or very large introns), the top and bottom 20% of the data were removed. C) Disorder table: the chart shows the absolute number of GFP-trap hits documented in disordered and non-disordered protein regions along with the number of 'misses' for each (the introns in the same genes not hit by a protein trap). [file 1471-2164-10-249-S2.pdf]
